# ISCEV standard pattern reversal VEP development: paediatric reference limits from 649 healthy subjects

**DOI:** 10.1007/s10633-023-09952-9

**Published:** 2023-11-08

**Authors:** Dorothy A. Thompson, Eszter Mikó-Baráth, Sharon E. Hardy, Gábor Jandó, Martin Shaw, Ruth Hamilton

**Affiliations:** 1 The Tony Kriss Visual Electrophysiology Unit, Clinical and Academic, Department of Ophthalmology, Great Ormond Street Hospital for Children NHS Trust, London, UK; 2 UCL Great Ormond Street Institute for Child Health, University College London, 30 Guildford Street, London, UK; 3 Institute of Physiology, Medical School, University of Pécs, Pécs, Hungary; 4 University College London Hospitals NHS Foundation Trust, London, UK; 5 Department of Clinical Physics and Bioengineering, NHS Greater Glasgow and Clyde, Royal Hospital for Children, Glasgow, UK; 6 College of Medical, Veterinary and Life Sciences, University of Glasgow, Glasgow, UK

**Keywords:** Reference data, Normative data, Brain development, Neural development, Maturation, Human

## Abstract

**Purpose:**

To establish the extent of agreement for ISCEV standard reference pattern reversal VEPs (prVEPs) acquired at three European centres, to determine any effect of sex, and to establish reference intervals from birth to adolescence.

**Methods:**

PrVEPs were recorded from healthy reference infants and children, aged 2 weeks to 16 years, from three centres using closely matched but non-identical protocols. Amplitudes and peak times were modelled with orthogonal quadratic and sigmoidal curves, respectively, and two-sided limits, 2.5th and 97.5th centiles, estimated using nonlinear quantile Bayesian regression. Data were compared by centre and by sex using median quantile confidence intervals. The ‘critical age’, i.e. age at which P100 peak time ceased to shorten, was calculated.

**Results:**

Data from the three centres were adequately comparable. Sex differences were not clinically meaningful. The pooled data showed rapid drops in P100 peak time which stabilised by 27 and by 34 weeks for large and small check widths, respectively. Post-critical-age reference limits were 87–115 ms and 96–131 ms for large and small check widths, respectively. Amplitudes varied markedly and reference limits for all ages were 5–57 μV and 3.5–56 μV for large and small check widths, respectively.

**Conclusions:**

PrVEP reference data could be combined despite some methodology differences within the tolerances of the ISCEV VEP Standard, supporting the clinical benefit of ISCEV Standards. Comparison with historical data is hampered by lack of minimum reporting guidelines. The reference data presented here could be validated or transformed for use elsewhere.

**Supplementary Information:**

The online version contains supplementary material available at 10.1007/s10633-023-09952-9.

## Introduction

All ISCEV Standards, including the VEP Standard, note the need for adequate reference data to support interpretation of visual electrophysiological recordings [1]. The reference interval is the most widely used medical decision-making tool [2], required to determine normality or otherwise of clinical parameters [3]. The gold standard for establishing reference intervals requires a sample of subjects selected from the reference population using pre-defined criteria to match the demographics of the patient population while excluding subjects with factors or disease likely to affect the parameter of interest [4]. Reference limits are then calculated using the nonparametric method, and the precision of each limit, usually its 90% confidence interval (CI), are calculated exactly and nonparametrically which requires a minimum of 120 reference data points [5]: larger samples may be needed to meet the further requirement that this precision is < 0.2 of the whole reference interval, where the reference interval is the distance between the lower and upper limits [6, 7]. These requirements should be met for each partition, i.e. each demographic variable such as sex which has a clinically significant effect on the parameter of interest [3].

Age is a continuously changing variable and the P100^1^ of the pattern reversal VEP (prVEP) changes markedly during infancy and childhood, its peak time being later in younger babies [8–15]. The P100 peak time changes reflect multiple maturational factors, for example, retinal maturation including foveal maturation, cortical changes and increased myelination. Derivation of age-related reference data typically employs a cross-sectional study design with ages suitably sampled for robust centile estimation and may require as many as 500 reference subjects [16]. Robust curve fitting allows for age compensation prior to outlier removal, and large samples enable separate fitting of upper and lower reference limits allowing the reference interval width to change with age [17, 18].

Given the time-consuming and costly nature of acquiring reference data, centres may choose to use external reference data from the scientific literature or commercial sources. Adopting external reference data for local use via the verification process [3, 19, 20] may require testing of only 20 local reference subjects: if external data require modification, this can be undertaken via the transference process [20, 21]. Transferring and verifying external reference datasets allows sharing of large, high-quality datasets and removes one obstacle from the process of delivering high quality visual electrophysiology testing.

The three purposes of this study were:

1. to compare retrospectively obtained paediatric reference datasets (binocular prVEP P100 parameters to large and to small checks) from three European centres, recorded using closely but not perfectly matched ISCEV Standard protocols;
2. to examine the effect of sex;
3. to establish reference limits and their confidence intervals, and present graphically and as formulae to facilitate verification and/or transference by other centres.

## Methods

### Subjects

All three centres recruited subjects specifically for the purpose of acquiring reference data for ISCEV Standard prVEPs. Studies were approved by local ethics boards, and all parents or guardians gave informed, written consent. Although some subjects were tested on more than one occasion, here we present data only from each subject’s first visit. Refraction was worn as necessary, but not formally checked as part of the visit. All three centres excluded children with a history of premature birth.

#### Royal Hospital for Children, Glasgow, UK (RHCG)

Infants and children were recruited and tested in 1997 as part of development of a steady-state VEP system for estimating acuity [22]; at the end of the session, transient prVEPs to multiple check widths were also recorded in descending size order. “Healthy children with normal eyesight” were recruited via local newspaper and radio adverts, aiming for ten subjects per age group in 2-month intervals up to 8 months, 4-month intervals up to 24 months, 1-year intervals up to 10 years, and a 5-year interval up to 15 years. A screening questionnaire was used to exclude children with poor general health or neurological or ophthalmic disease. Acuity was checked to be normal for age using age-appropriate tests binocularly and monocularly. Data were obtained from 180 subjects ranging in age from 5 weeks to 16 years old; of those, 52 were infants aged 5–52 weeks. Largely reflecting typical national demographics at the time, all children were White.

#### Great Ormond Street Hospital, London, UK (GOSH)

Subjects were recruited from neonatal wards and local health centres and tested from 2002 onwards to establish reference data for flash VEPs, flash ERGs and prVEPs to multiple check widths. The 50’ check width was presented first then larger and smaller check widths interleaved, using large check widths to encourage attention for subsequent smaller check widths. All children were free from known systemic ophthalmic or neurological conditions. The majority of children were White but data regarding race and ethnicity have not been retained. Data were obtained from 219 subjects ranging in age from 2 weeks to 16 years old; of those, 165 were infants aged 2–52 weeks.

#### University of Pécs, Medical School, Hungary (UPMS)

Subjects were recruited by letter inviting local new parents to participate, and were tested during 2007–20, many as full term controls for studies of binocularity and stereopsis [23, 24] following premature birth. Transient prVEPs to multiple check widths (120–7.5ʹ) were recorded. Children were excluded if they had strabismus, epilepsy, medications for any systemic conditions, maternal concern about fixing and follow, family history of inherited retinal disease, or perinatal time in neonatal intensive care or special care. Age-appropriate eye movement and fixation were inclusion criteria. Data were obtained from 272 subjects ranging in age from 2 weeks to 6.5 years old; of those, 242 were infants aged 2–52 weeks. All participants were White.

### VEP recordings

All three centres recorded binocular prVEPs from Oz referred to Fz in response to high contrast black and white reversing (alternating) checkerboards. Only data for check widths matching the ISCEV standard (within specified tolerance of 60’ and 15’) are described here, subsequently called “large check widths” and “small check widths”. Notch filters were not used. Details of stimulus and acquisition parameters are given in Table 1. Impedance criteria were less stringently followed than stipulated in the VEP standard because of the need to retain infant or child cooperation, and compensatory signal quality checks were employed.

**Table 1:** Pattern reversal VEP stimulus and acquisition parameters for the three centres, and as required by the most recent ISCEV Standard [1]

	RHCG	GOSH	UPMS	ISCEV standard
Sampling rate (kHz)	1	1	0.96	≥ 1
Bandwidth (Hz)	1–100 (1st order analogue)	0.3–300	0.5–250	At 3 dB; ≤ 1 and ≥ 100
Sweep duration (ms)	1024	400 (1/s) or 285 (3/s)	1066	≥ 250
Pre-stimulus duration (ms)	0	15	0	Not stated
# channels; location	3: Oz–Fz, RO–Fz, LO–Fz	4: Oz–Fz, RO–Fz, LO–Fz and inion–Fz	1: Oz–Fz	1: Oz–Fz
Impedance (kΩ)	aim < 10, matched within 3	< 10: aim 5, closely matched	Signal quality check	< 5, matched within < 1
Total # sweeps	30–120	30–100	≥ 150	≥ 50 (OK to do fewer)
# Replications	≥ 2	≥ 2	2 and/or statistical technique	≥ 2
Stimulus monitor (all cathode ray tube, CRT)	IBM 14XG, 14″, 76 Hz refresh, 800 × 600 pixels	NEC MultiSync 4PG, 27″, 60 Hz refresh, 1024 × 768 pixels	Samsung 957MB, 19″, 60 Hz refresh, 1024 × 768 pixels	
Reversals per second	1.1	1 (< 8 wks); 3 (≥ 8 wks)	3.75	1.8–2.2
Trigger point	Top left (corrected, + 7.1ms)	Mid-screen	Mid-screen	Mid-screen*
Large check width (ʹ)	60	50	60	48–72
Small check width (ʹ)	12	12.5	15	12–18
Viewing distance (cm)	45	100	50	Typically 50–150
Field size, horizontal × vertical (°)	33.4 × 25.1	28 × 21	30 × 40	≥ 15
Aspect ratio	4:3	4:3	4:3	≤ 4:3
Michelson contrast (%)	95	~ 98	~ 95	≥ 80
Mean luminance (cd/m^−2^)	60	60–80	54.4	40–60
Fixation mark	Small red square	Coloured circle ~ 5–10′	Transparent image 1–3°	
Room lighting	Ordinary	Dim/off	Dark	Dim or ordinary

*Earlier versions of the VEP Sstandard required peak times to be measured “from the onset of the stimulus” [26]

#### Royal Hospital for Children, Glasgow, UK (RHCG)

VEPs were recorded using a custom-built system [25] and adhered to the VEP Standard current at the time of recording [26]. Acquisition was triggered at the top-left point of the screen refresh rather than at the midpoint specified in the current standard [1], as required by the 1995 VEP Standard “peak times should be measured from the onset of the stimulus” [26]: peak time values have been adjusted to emulate triggering acquisition at the screen refresh mid-point by adding 7.1 ms. The reversal rate of 1.1 s^−1^ was slower than permitted in the current standard (1.8–2.2 rev s^−1^ [1]), but matched the requirement (“less than 2 stimuli per second”) of the 1995 standard.

#### Great Ormond Street Hospital, London, UK (GOSH)

VEPs were recorded using a Diagnosys E3 system (Diagnosys LLC, Lowell, MA, USA) and acquisition triggered at the midpoint of the screen refresh. Reversal rates of 1 s^−1^ and sweep durations of 400 ms were employed for only the very youngest infants (< 8 weeks). For older infants and children, the reversal rate of 3 s^−1^ was faster than stipulated in the current standard [1] to optimise child engagement via shorter recording times.

#### University of Pécs, Medical School, Hungary (UPMS)

Signals were collected and processed with CED 1401 Power (Cambridge Electronic Design Limited, Cambridge, UK) data acquisition equipment with a custom-built signal amplifier. CED Spike2 software v6 was used for stimulus generation, data acquisition and analysis. A reversal rate of 3.75 s^−1^, faster than stipulated in the current standard [1] but which still evokes a transient waveform, was used to shorten recording times and maintain engagement.

### Data analysis

For two centres (GOSH and RHCG), the presence of a reproducible, prVEP-like waveform was judged by an expert observer. The third centre (UPMS) used the same technique for a minority (≈10%) of recordings and in every case employed a statistical technique [27]: records were divided into 1.066 s non-overlapping epochs and a fast Fourier transformation applied to each epoch: Fourier components at the reversal rate (i.e. 3.75 Hz, considered the fundamental frequency) were subjected to a *T*^2^_circ_ statistic at a *p* = 0.01 significance level. The *T*^2^_circ_ statistic is generally used for steady-state evoked potentials and was used for analysis of both the transient prVEPs described here and also the steady-state VEPs recorded as part of a binocularity investigation [23, 24].

For all centres, reproducible or significant prVEPs had amplitude and peak time of P100 determined by manual cursoring of each subject’s grand average of the significant response. P100 peak time was measured from stimulus onset as described above, and P100 amplitude was measured from N75 if evident, or from baseline if not.

Inspection of amplitude (*A*) data plotted versus age (weeks), both after natural logarithmic transformation, showed two clear clusters of data similar in magnitude. An orthogonal quadratic model under those transformations was chosen:

1 $$\mathrm{ln}\left({A}_{\mathrm{P}100}\right)= \frac{a\left(\left(\mathrm{ln}\left(x\right)-d\right)\left(\mathrm{ln}\left(x\right)-e\right)-\frac{g}{f}\right)}{\sqrt{h}}+\frac{b\left(\mathrm{ln}(x)-e\right)}{\sqrt{g}}+c$$

where *A*_P100_ is the centile value (μV), *x* is age (weeks), *d*, *e*, *f* and *g* are constants which describe the curve for a particular check width and *a*, *b* and *c* are constants which describe the relevant centile of that curve. The amplitude centiles look nonlinear after back-transformation and are described by:

2 $${A}_{\mathrm{P}100}=\mathrm{exp}\left(\frac{a\left(\left(\mathrm{ln}\left(x\right)-d\right)\left(\mathrm{ln}\left(x\right)-e\right)-\frac{g}{f}\right)}{\sqrt{h}}+\frac{b\left(\mathrm{ln}(x)-e\right)}{\sqrt{g}}+c\right)$$

A sigmoid curve was fitted to peak time data [24]:

3 $${t}_{\mathrm{P}100}=\left[\frac{b-a}{\left(1+\mathrm{exp}\left[\frac{{x}_{0}-x}{\mu }\right]\right)}\right]+a$$

where *t*_P100_ is the centile value (ms), *b* is the upper asymptotic value of the centile at age = 0 (ms), *a* is lower asymptotic ‘adult’ value of the centile (ms), *x*_0_ is age at the midpoint of the slope (weeks), *x* is age (weeks), and *μ* is a dimensionless constant proportional to the gradient of the slope. Nonlinear quantile Bayesian regression was used to estimate centiles and their bootstrapped (*n* = 40,000) 90% CIs. Although it is generally true that a late and/or small prVEP P100 represents abnormality, early and/or large pattern VEP P100s can also be associated with pathology [28, 29]. Two-sided rather than one-sided limits were therefore calculated using conventional 2.5th and 97.5th centiles, i.e. enclosing 95% of data values. This choice accepts a 5% false positive chance for every parameter assessed.

To investigate the extent of similarity of data from the three centres, three processes were followed. Firstly, data identifiable by centre were visually inspected for conspicuous differences. Secondly, peak time data from two centres were combined and used to construct 2.5th and 97.5th centile estimates: the number of points from the third centre lying outwith this reference interval were counted, similar to standard verification procedures [4, 19, 21]. This was repeated for all three centre combinations. Thirdly, the median (50th centile) and its bootstrapped 90% CI was estimated for each centre and inspected for separated or overlapping CIs of centre median lines. This last technique was also used to investigate the effect of sex.

A ‘critical age’ was calculated for each check width, i.e. the age at which P100 peak time ceased to shorten, defined as the age at which the upper (97.5th) percentile dropped to within 1% of its asymptotic value. Peak time reference limits were calculated for data from infants and children older than these critical ages using the nonparametric percentile method [4] and compared with asymptotic values obtained from Eq. (3). Amplitude reference limits were calculated from all data using the same Clinical and Laboratory Standards Institute (CLSI) process and compared with values obtained from Eq. (2).

Modelling was undertaken using R (R Core Team, 2020) and RStudio (RStudio Team, 2020) [30]. Additional analyses used MedCalc® Statistical Software version 20.115 (MedCalc Software Ltd, Ostend, Belgium; https://www.medcalc.org; 2022).

## Results

Absent (non-reproducible or non-significant) prVEPs to large check widths were noted for 14/318 children (4%) at UPMS, aged 7 months on average (sd 3 months), of whom half were girls: all had prVEPs present to a different check width (120’, 30’ or 15’). At RHCG, 1/180 children had an absent prVEP to large checks, a 1 month old boy who had prVEPs present to larger check widths; no prVEPs attempted to smaller checks. For small check widths, prVEPs were absent for 46/318 (14%) children at UPMS, aged 4.5 months on average (sd 3 months), of whom half were girls: all had prVEPs present to 60’ check widths, and no prVEPs were attempted to smaller checks. At RHCG, 4/94 (4%) children (two boys and two girls aged < 7 months; all had prVEPs present to larger check widths and no prVEPs were attempted to smaller checks. Data concerning any absent responses were not retained by GOSH. The final dataset therefore comprised large check width prVEPs from 649 infants and children and small check width prVEPs from 403 infants and children (Table 2).

**Table 2:** Number of subjects providing data by centre and by sex

		RHCG	GOSH	UPMS	Total
Large checks	Female (*n*, %)	95 (56%)	129 (60%)	126 (48%)	350 (54%)
	< 1 yr (*n*, %)	46 (27%)	161 (75%)	236 (88%)	443 (68%)
	Total *N*	169	214	266	649
Small checks	Female (*n*, %)	51 (57%)	74 (62%)	100 (52%)	225 (56%)
	< 1 yr (*n*, %)	6 (7%)	89 (75%)	175 (90%)	270 (67%)
	Total *N*	90	119	194	403

### Comparability between centres

**Fig. 1:**
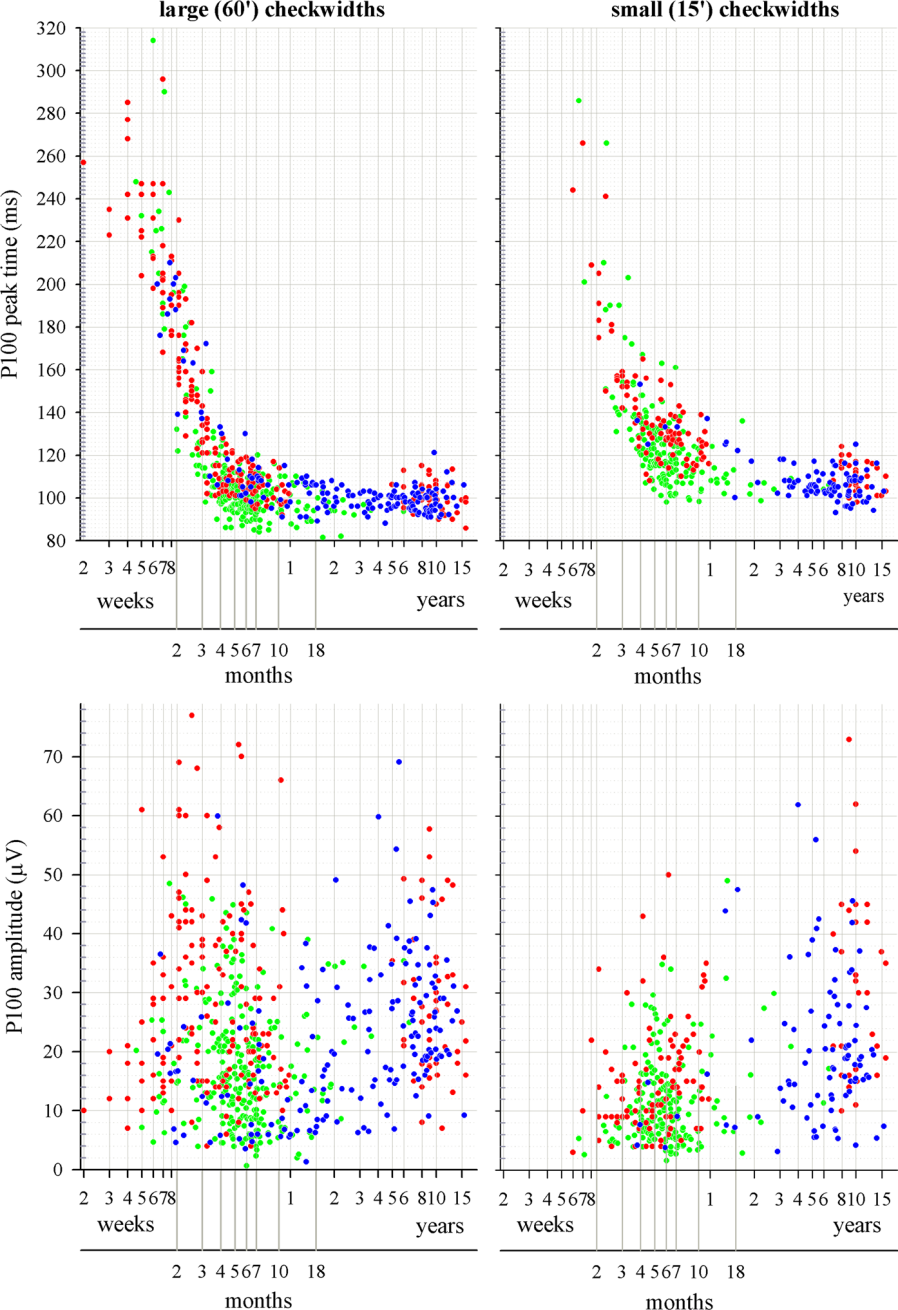
Peak times (upper panels) and amplitudes (lower panels) of P100 versus age. Large check width data are shown in left hand panels, small check width data are shown in right hand panels. Red, GOSH. Blue, RHCG. Green, UPMS

Data were largely comparable between the three centres by inspection (Fig. 1) but suggested slightly faster P100 peak times to large check widths from UPMS. For peak times, an adequately small proportion of data points from each centre fell outwith reference limits constructed for combined data from the two other centres (Table 3) and outliers were distributed symmetrically above and below limits except for large check width P100 peak times for UPMS, which were generally slightly faster between about 15–25 weeks than for GOSH and RHCG data combined. This was also evident when comparing overlaps of the CIs of each centre’s peak time median values (Figure S1), with UPMS large check width peak times not overlapping for a small age range of about 15–18 weeks. However, the absolute size of the differences between medians (< 10 ms) was small relative to the overall data dispersion at that age (> 50 ms). Peak time asymptotic values (i.e. peak times after the critical ages) showed only small inter-centre differences in median values, being 103 ms, 100 ms and 98 ms for large check widths, and 117 ms, 106 ms and 111 ms for small check widths, for GOSH, RHCG and UPMS, respectively. Given that 1) data distribution with age differed between centres, 2) centre differences were not reproduced across both check widths, and 3) the relatively small size of differences, data were considered adequately comparable to combine into a single reference dataset.

**Table 3:** Number of peak time data points from one centre falling outwith reference limits constructed from the other two centres

	RHCG data relative to GOSH and UPMS data	GOSH data relative to RHCG and UPMS data	UPMS data relative to RHCG and GOSH data
Large checks	4/169 (2.4%)	14/214 (6.5%)	29/266 (10.9%)
Small checks	7/90 (7.8%)	5/119 (4.2%)	9/194 (4.6%)

### Effect of sex

Data from all three centres stratified by sex were largely comparable by inspection (Figure S2). No peak time differences were seen to large check widths (CIs overlapped for male and female 50th centiles). Girls had slightly faster P100s to small check widths between about 8 and 18 weeks, but the difference was small (< 10 ms) compared with data dispersion at that age. Asymptotic differences in median values were also very small, being 99.7 ms and 100 ms for large check widths, and 109 ms and 110 ms for small check widths, for girls and boys, respectively. Given that inter-sex differences were small and not clinically meaningful compared with very large differences with age and given the loss of precision which would result from halving the dataset (partitioning into male and female datasets), separate male and female reference intervals were not considered to be justified.

### Reference intervals

Parameters for use in Eqs. (2 and 3) to derive upper and lower reference limit values for any age are given in supplementary material (Tables S1, S2). Look up values are given in Tables 4 and 5.

**Table 4:** Amplitude CLSI derived reference limits for all ages (2 weeks to 16 years), lower (2.5th percentile) – upper (97.5th percentile) for P100 of the pattern reversal VEP. Each limit's 90% confidence interval is given in brackets

Large check width (50ʹ/60ʹ) (μV)	Small check width (12ʹ/15ʹ) (μV)
5–57 (4–5) and (49–60)	3.4–45 (3–4) and (42.5–54)

#### Large check widths (50ʹ/60ʹ)

##### Amplitude

**Fig. 2:**
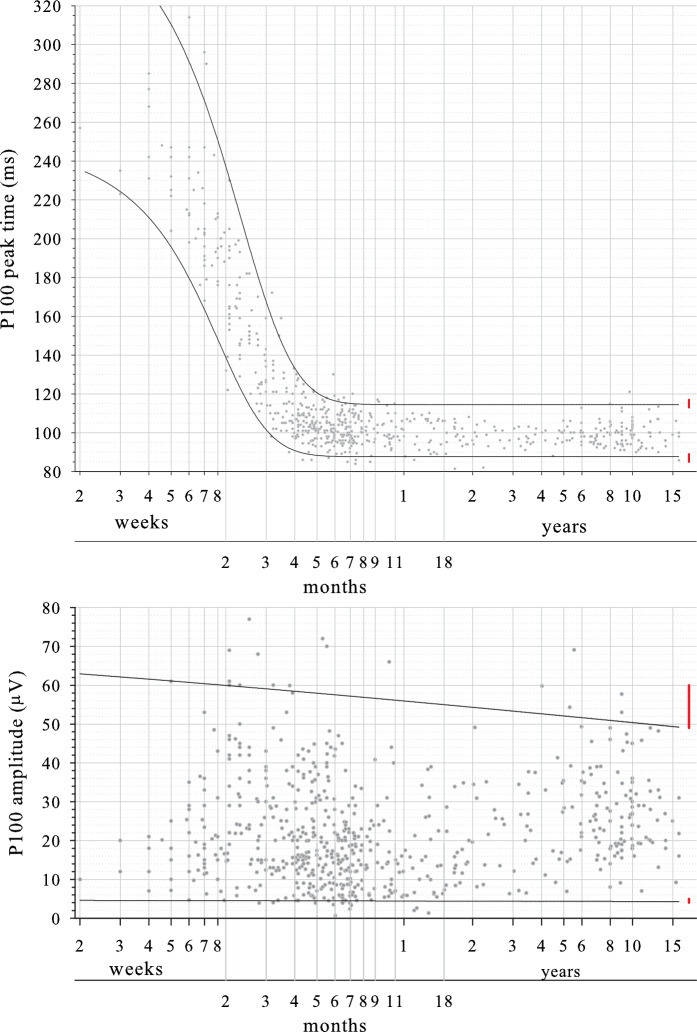
Reference limits for P100 to large check widths. Upper panel, peak time. Lower panel, amplitude. Upper reference limit is 97.5th percentile, lower reference limit is 2.5th percentile. Age is shown on a logarithmic scale. Red bars at the oldest ages are reference limits (± 90% CI) derived from post-critical age data (27 weeks) for peak time and for all ages for amplitude using the nonparametric percentile method approved by CLSI C28-A3 guideline

Amplitudes varied markedly at all ages and were skewed to lower values (Fig. 2, lower panel). A wider CI for the upper limit than the lower limit reflects this skew (Figure S3). Limits showed only minor changes with age. For large check widths, the lower limit is around 4 μV at all ages, while the upper limit drops from around 60 μV at 2 months to around 50 μV by teenage years. Limits calculated from all data points (i.e. not considering changes with age) using the CLSI nonparametric percentile method were 5 μV and 57 μV (Table 4; red lines in Fig. 2 lower panel). Limit precision (i.e. 90% CI) was adequately narrow relative to the whole reference interval (< 0.2) for the lower limit (0.02) but not quite for the upper limit (0.21).

##### Peak time

There was a rapid drop in peak time during infancy which stabilised during the second six months of life (Fig. 2, upper panel). Wider CIs at younger ages at least partially reflect the smaller number of data points (Figure S4). The critical age was 27 weeks, i.e. very close to 6 months old. For the 338 subjects older than this critical age of 27 weeks, CLSI-derived reference limits for peak time (90% CIs) were 87 ms and 115 ms (Table 5; red lines in Fig. 2 upper panel). Both limits met the criterion that their precision (i.e. 90% CI) was less than 0.2 of the whole reference interval: peak time lower limit 0.14; peak time upper limit 0.14.

**Table 5:** Look up table for peak time reference limits for P100 of the pattern reversal VEP. Numbers are lower (2.5th percentile) – upper (97.5th percentile) limits. For the two post-critical age CLSI derived limits, values in brackets are the 90% CIs of each limit

Age range	Large check width (50ʹ/60ʹ) peak time (ms) lower–upper	Small check width (12ʹ/15ʹ) peak time (ms) lower–upper
< 4 weeks	236–358	253–441
4–5 weeks	203–319	212–385
5–6 weeks	188–301	198–362
6–7 weeks	171–281	185–339
7–8 weeks	156–261	173–316
8–9 weeks	141–241	163–294
9–10 weeks	129–222	154–274
10–11 weeks	118–204	146–256
11–12 weeks	110–188	138–239
12–13 weeks	104–174	132–224
13–15 weeks	98–157	125–204
15–17 week	93–141	117–184
17–19 week	90–131	111–170
19–21 week	89–124	107–159
21–23 week	88–120	104–152
23–25 week	88–118	102–147
25–27 week	88–116	100–144
27–29 week		99–141
29–31 week		98–140
31–34 week		98–139
Post-critical age CLSI derived limits (90% CIs)	87–115 (85–89) and (113–117)	96–131 (93–98) and (125–139)

#### Small check widths (12ʹ/15ʹ)

##### Amplitude

**Fig. 3:**
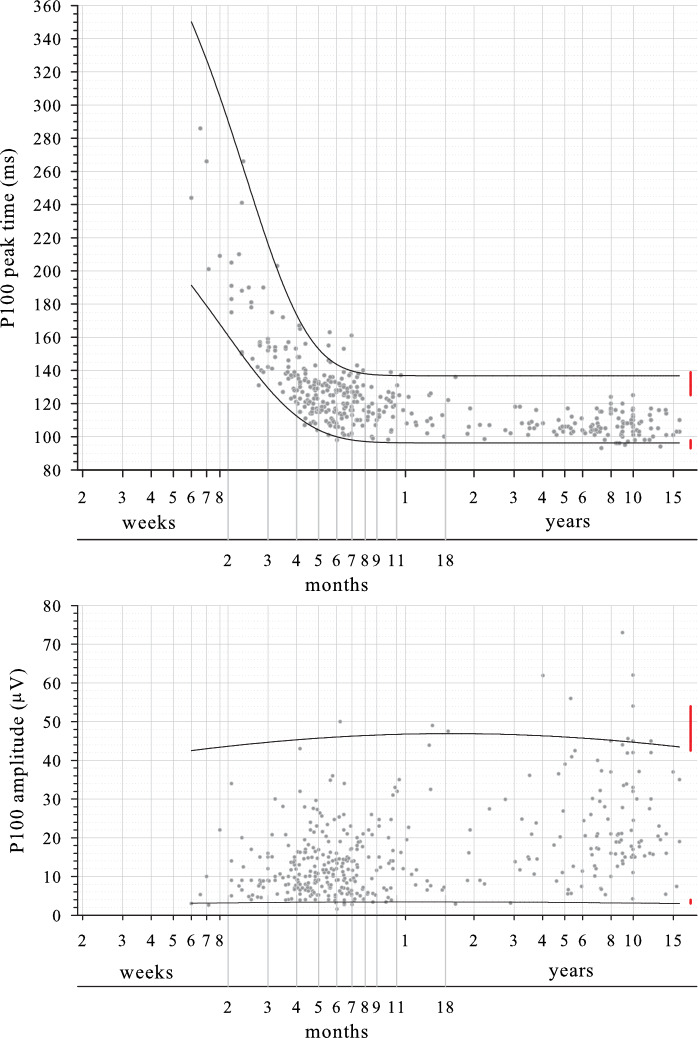
Reference limits for P100 to small check widths. Upper panel, peak time. Lower panel, amplitude. Upper reference limit is 97.5th percentile, lower reference limit is 2.5th percentile. Age is shown on a logarithmic scale. Red bars at the oldest ages are reference limits (± 90% CI) derived from post-critical age data (34 weeks) for peak time and for all ages for amplitude using the nonparametric percentile method approved by CLSI C28-A3 guideline

Amplitudes varied markedly, but limits changed little with age being around 3 μV and 45 μV at all ages (Fig. 2, lower panel). As for large check widths, amplitudes were skewed to lower values with the wider CI for the upper limit than the lower limit reflecting this exponential distribution (Figure S3). Limits calculated from all data points (i.e. not considering changes with age) using the CLSI nonparametric percentile method were 3.5 μV and 56 μV (Table 3; red lines in Fig. 3 lower panel). Limit precision (i.e. 90% CI) was adequately narrow relative to the whole reference interval (< 0.2) for the lower limit (0.04) but not for the upper limit (0.54).

##### Peak time

Peak time decreased rapidly during infancy and stabilised towards the end of the first year of life (Fig. 3 upper panel): as for large checks, CIs were wider at younger ages (Figure S4). The critical age for small checks was 34 weeks. For the 168 subjects older than this critical age of 34 weeks, CLSI-derived reference limits for peak time were 96 ms and 131 ms (Table 5; red lines in Fig. 3 upper panel). While the lower limit met the criterion that its precision (i.e. 90% CI) was less than 0.2 of the whole reference interval (peak time lower limit 0.14), the upper limit did not (peak time upper limit 0.40).

## Discussion

These data showed that—despite some methodology differences—prVEP reference data from three centres could justifiably be pooled to create a single large dataset. Partition by sex was not justified because of small inter-sex differences. ISCEV Standard large check width prVEP peak time reduced dramatically over the first 27 weeks of life and thereafter was stable and adult-like, while amplitude was highly variable. Small check width prVEP peak times were later than for large checks and showed a similar but less dramatic reduction in peak time which stabilised by 34 weeks of age; amplitude was again highly variable. The ages at which peak times stabilise match closely the age when optic nerve fibres near the globe are virtually all myelinated [31] and suggest the end of a critical period, also known as a developmental window. These periods can be determined with high precision, providing important information for further studies on development.

These findings fit with current understanding of the prVEP, namely that peak time is the parameter of primary interest while, due to inter-individual variability, amplitude is mostly relevant to inter-visit or inter-ocular assessment of an individual.

While we observed a few instances of non-reproducible prVEPs in the youngest infants, we note that these were volunteer subjects undergoing long research protocol where researchers were reluctant to persist with testing a subject who was tiring. In our collective clinical experience, the prVEP is robustly recordable from the earliest weeks of life, often even when infants show less than normal visual behaviour.

The confidence intervals of the asymptotic reference limits derived from quantile regression of the entire dataset overlapped with confidence intervals of limits calculated for only subjects older than the critical ages, reassurance that our methods are appropriate. Despite the large sample size, the criterion that a limit’s precision (90% CI) should be less than 20% of the entire reference interval was not met for the upper limits for amplitude, nor for the upper limit of peak time for small checks.

The data presented here were all recorded using CRT stimulus monitors with typical refresh rates of 60–80 Hz, i.e. electron beam typical scan time (repeated horizontal scans from top left to bottom right) of 12–17 ms. PrVEP acquisition is triggered at ‘time zero’, usually defined as either the time when the scan is at the top-left of the screen refresh, or when it reaches the mid-point of the screen refresh. The difference between a top-left and a mid-screen trigger adds 6–8 ms to P100 peak time. The earliest VEP Standard required that peak times should be measured “from the onset of the stimulus” [26], which has been interpreted as a top-left trigger point. Subsequent revisions made no stipulation [32, 33]. The current Standard notes that screen refresh rates vary and requires that peak times be measured with time zero defined as the mid-point of the screen refresh [1]. In the absence of a specific requirement to state time zero relative to screen refresh when reporting prVEPs, this considerable offset may not be known or acknowledged. CRTs are no longer made. Liquid crystal displays (LCDs) produce luminance artefacts which may generate a flash VEP in the absence of a prVEP unless specific compensation is made, which may include reducing pattern contrast [34, 35]. Organic electroluminescence (OLED) screens may introduce delay [36]. If the CRT reference data presented here are adopted for use with prVEPs recorded with different display technologies, specific numerical adjustments should be incorporated based upon timing differences which can be estimated, along with likely error, by comparing prVEPs from a CRT-based system and the alternative display technology.

**Fig. 4:**
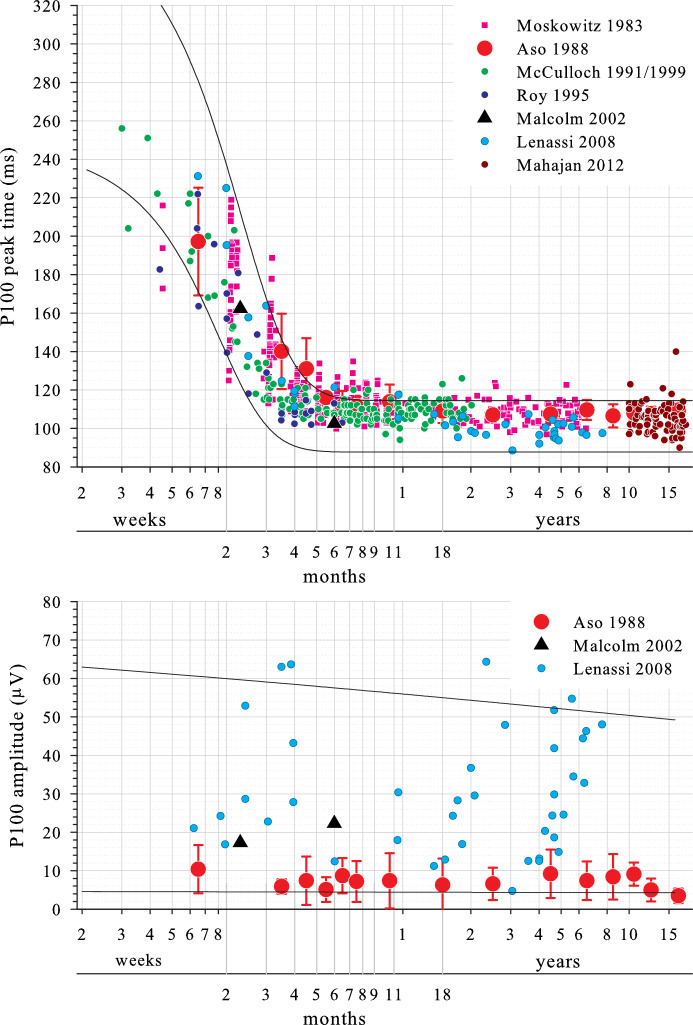
Pattern reversal VEP P100 data to large check widths from comparable studies. Upper panel, peak time. Lower panel, amplitude. Colour data are from other studies as identified in the legend. Solid lines are reference limits derived from data in the current study

There are relatively few similar studies of infants and children which post-date the first ISCEV Standard and where binocular stimulation was used [13–15]. Studies which pre-date the first ISCEV Standard are nonetheless important, particularly those with substantial sample sizes [8–10] or which encompass the ages of most rapid change [8–10, 12] (Table 6). Considering large check width P100 amplitude (Fig. 4, lower panel), some data correspond well with limits derived from the current study [13, 14]. One study found overall smaller prVEPs, particularly at older ages [9]: data from this study were available only as mean and one standard deviations, summary values which do not represent well the highly asymmetric amplitude data typical of the prVEP P100. Furthermore, the authors do not give luminance values; lower luminance may have resulted in lower amplitudes. For small check widths, comparable P100 amplitude data were available from only one study [13] and fell within reference limits defined in the current study.

**Table 6:** Summary of stimulus and acquisition parameters for comparable studies

Study	Subjects	Field size (°)	Test distance (cm)	Check width (min of arc)	Reversal rate	Stimulus type	Trigger	Mean luminance (cd/m^2^)	Contrast (%)	Active electrode site	Reference electrode site	Bandpass (Hz)	Epoch duration (ms)	# averages; # replications
Moscowitz [8]	*N* = 320 1mo–5 yr	15 × 18	75	60’/15’	1.88 alt s^−1^ (0.94 Hz)	“TV monitor”	ns	78	84	1 cm above inion	Ear	1–35	ns	32, 64 or 128; 1
	*N* = 119 2–6mo	11 × 14	100	48’/12’	1.88 or 3.75 alt s^−1^ (1.88 Hz)							1–35 or 1–50		
Aso [9]	*N* = 141 1mo–19 yr	32 × 25	60	50’	2 alt s^−1^ (“reversed every 1/sec”)	“Television screen”	ns	ns	“black and white”	Oz, O1, O2	Cz	0.53–120	500	32 (< 2 yr), 64; 2
McCulloch [10]	*N* = 161 3wk–2 yr	ns	75	60′ 15’	phase reversal 1.88 alt s^−1^	“Video screen”	“Each phase reversal”	55	95	1 cm above inion	Earlobe	0.1–30	400	20–40; 2
Roy [12]	*N* = 24 1–6mo	30 × 30	70	60′ 15’	reversed at 1.3 alt s^−1^	CRT	ns	ns	“black and white”	Oz	Earlobe	1–100	500; 50 pre-stimulus	50–100; 1
Malcolm [13]	*N* = 50 10 and 26wk	30 × 24	43	60′ 12’	2.2 rev s^−1^ (1 Hz)	CRT	Top-left (7.1 ms < midscreen)	60	> 90	Oz	Fz	0.5–100	500	30–60; 2
Lenassi [14]	*N* = 41 1.5mo–7.5 yr	28 × 22	100	50’	3 Hz	“Television screen”	ns	100	90	Oz, O2’, O1’	Fz	0.3–300	300, 10 pre-stimulus	50–100; 2
Mahajan [15]	*N* = 81 10–16 yr	23	100	50′ 15’	2 Hz	“Projected on computer screen”	ns	60.7	85	Oz + 29 others	Mastoid	1–100	ns	200; 1

See Figs. 4 and 5

*alt* Alterations; *ns* Not stated

For large check width P100 peak times (Fig. 4, upper panel), some studies agreed closely with the current data [12–15]. Two studies agreed well for infants younger than about 6 months but found later P100s for older infants and children [8, 10], both of which used stringent amplifier high frequency filters with low-pass cut-offs at 35 or 50 Hz [8] and at 30 Hz [10], and pre-dated the first VEP Standard which required a minimum of 100 Hz [26]. For adult prVEPs, P100 is prolonged by 7 or 8% for an analogue 30 Hz low-pass filter relative to a 100 Hz low-pass filter [37]: this delay is unlikely to be incurred for the later, broader prVEPs with relatively low frequency content seen in young infants, but will start to affect the faster, high frequency content of maturing prVEPs from older children, prolonging their P100 relative to those described in the current study. Furthermore, although not always explicitly stated, it is most likely that a CRT stimulator was used. The screen trigger point for data acquisition is unknown for all but one study, and it is possible that some or even all of these published data represent a screen top-left trigger for acquisition, adding a systematic 6–8 ms ‘delay’ relative the current data.

**Fig. 5:**
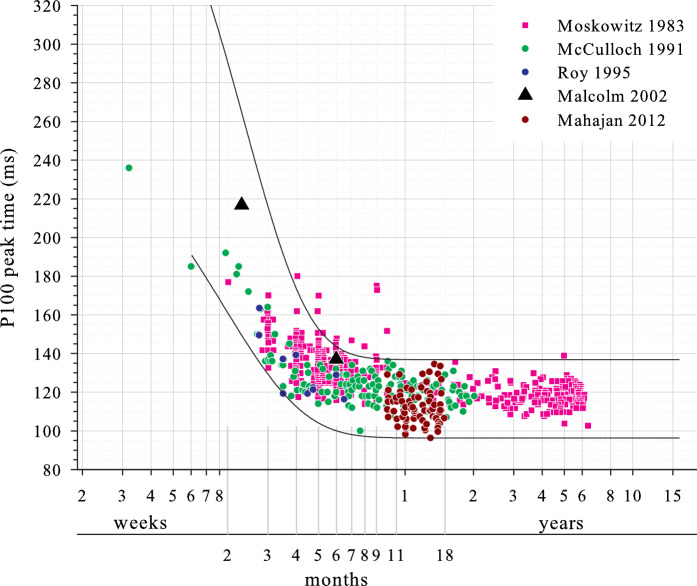
Pattern reversal VEP P100 data to small check widths from comparable studies (peak times). Colour data are from other studies as identified in the legend. Solid lines are reference limits derived from data in the current study

For small check width P100 peak times (Fig. 5), all studies with available data agreed closely with the current data [8, 10, 12, 13, 15]. However, at around 4–12 months of age, the spread of P100 peak times for the two largest studies tended to be distributed towards the upper limit of the current study’s reference limits [8, 10], perhaps related to their more stringent amplifier low-pass cut-offs and/or potentially different screen trigger point for data acquisition as discussed previously. It is unclear why these effects, if in force, are less for small check width VEPs than large check width VEPs.

Overall, the good concordance of the data presented here with previously published data establishes the robustness of data acquired according to the stringent international Standard [1] and reinforces the tremendous clinical benefit of Standards in enabling high comparability between centres [38, 39]. Difficulties with comparing across studies due to missing methodological data highlights the need for minimum reporting guidelines in visual electrophysiology [40].

### Sex

We found no amplitude differences by sex and only minimally faster peak times for girls at a limited age range (about 8–18 weeks) with no clinically meaningful differences in asymptotic peak times. Other studies have conflicting findings regarding the significance of any difference, dependent on subject age and sample size. Both larger P100 amplitudes in females [41–43] and no sex difference [13] have been reported. In childhood, female prVEPs are often reported to be a few milliseconds faster than male prVEPs (2 or 3 ms at 4–11 years [9]; 2 ms at 11 years [41]; 8 ms at 10 weeks and 16 ms at 6 months [13]), but no difference is also described [42, 44]. Both female sex and smaller head size appear to contribute to faster prVEP peak times [13, 41].

### Monocular vs binocular

VEPs described here were all collected with binocular viewing, although monocular stimulation is stipulated as standard [1]. While monocular prVEPs are routinely recorded in paediatric visual electrophysiology centres, there are occasions when they are not feasible (e.g. a child not tolerating occlusion) or not indicated (e.g. a child with no inter-ocular differences, or a child where assessment of their binocular visual pathway function is required). Therefore, binocular prVEP reference data are essential for safe and high-quality interpretation of paediatric patient prVEPs. Several studies have investigated monocular/binocular differences in healthy paediatric [9] or adult subjects [45]. Monocular P100 amplitudes are typically smaller, but can be anything from 50 to 100% of a binocular prVEP [45–48]. Monocular P100 peak times are typically 0–4 ms later than binocular P100s [9, 45, 47, 49, 50]. In the absence of separate monocular prVEP reference data, the binocular data presented here could be used cautiously to interpret monocular prVEPs, bearing in mind these clinical ‘rules of thumb’.

### Recommended process for centres wishing to adopt these reference limits

Note all data presented here were acquired from unsedated, awake infants and children and are therefore very unlikely to be applicable to prVEPs recorded under anaesthesia: as with the ERG [51], the prVEP is altered or even extinguished [52] by anaesthetic agents. The process of ensuring that a reference interval established elsewhere, such as the data published here, can be adopted locally with reasonable confidence, is known as verification (or validation) of a reference interval [3]. The following steps are required:

1. Initial verification: documented assessment of the reference dataset published here (the ‘primary reference interval’) relative to local protocols, i.e. demographic variables, method of estimating the reference limits, original test procedures: if these factors are subjectively judged to be comparable with the adopting centre’s test methods and patient population, then adoption is validated.
2. Further verification is usually necessary, particularly if not all required details of the reference interval are available. The adopting centre recruits 20 local reference subjects who satisfy exclusion and partition criteria: if no more than two local reference data points fall outside the primary reference interval, that interval can be considered acceptable for local use.
3. If three or four data points fall outside the primary reference range, a further 20 local subjects should be recruited and tested; if no more than two reference data points from this second local sample group fall outside the primary reference interval, the interval can be considered acceptable for local use.
4. Otherwise, a re-examination of test protocols should be considered, along with the possibility that the local patient population is substantially different to the reference subjects contributing to the primary reference sample.
5. This simple check is vulnerable to error for skewed distributions or variance differences between primary and local samples. The primary reference dataset is available [53], so comparisons using Mann–Whitney U, Siegel–Tukey or Kolmogorov–Smirnov are more sensitive and specific [2].
6. For greater accuracy in deciding the acceptability of a primary reference dataset, for example, where there is a particular local need for accuracy, larger numbers of local reference subjects should be tested.

## Conclusions

These data show that reference data collected using ISCEV Standard protocols are likely to be amenable to combining into larger datasets, with resultant better precision of reference limits and therefore increased diagnostic power. This is seen even with the current datasets, where some deviations in protocol, or variations within the tolerances of the ISCEV VEP Standard, nonetheless produced comparable reference datasets [53].

## Supplementary Information

Below is the link to the electronic supplementary material.

Supplementary file1 (PDF 275 KB)

## Footnotes

1 P100 is the conventional nomenclature for the prVEP peak, P for positivity, 100 as the typical adult value in milliseconds. Since its peak time can vary greatly beyond 100 ms, some authors have used the term P1 (first positive wave) for the same peak.

## Declarations

### Statement on the welfare of animals

The study involved no research on animals.
