# Supplementary material for: ISCEV standard pattern reversal VEP development: paediatric reference limits from 649 healthy subjects
Source: Doc Ophthalmol. 2023 Nov 8;147(3):147–64. doi: 10.1007/s10633-023-09952-9 (PMC10638119; doi:10.1007/s10633-023-09952-9)
Supplement: Supplementary file 1 — Supplementary file1 (PDF 275 KB) [file 10633_2023_9952_MOESM1_ESM.pdf]

Supplementary material – amended November 2024: Table S2, small checkwidth, lower percentile, value of μ is -5.77 (previously incorrect as 5.77).

ISCEV Standard pattern reversal VEP development: paediatric reference limits from 649 healthy subjects,

Documenta Ophthalmologica, Dorothy A. Thompson, Eszter Mikó-Baráth, Sharon E. Hardy, Gábor Jandó, Martin Shaw, Ruth Hamilton. Corresponding author Dorothy A. Thompson, The Tony Kriss Visual Electrophysiology Unit, Clinical and Academic, Department of Ophthalmology, Great Ormond Street Hospital for Children NHS Trust, London, UK; dorothy.thompson@ucl.ac.uk.

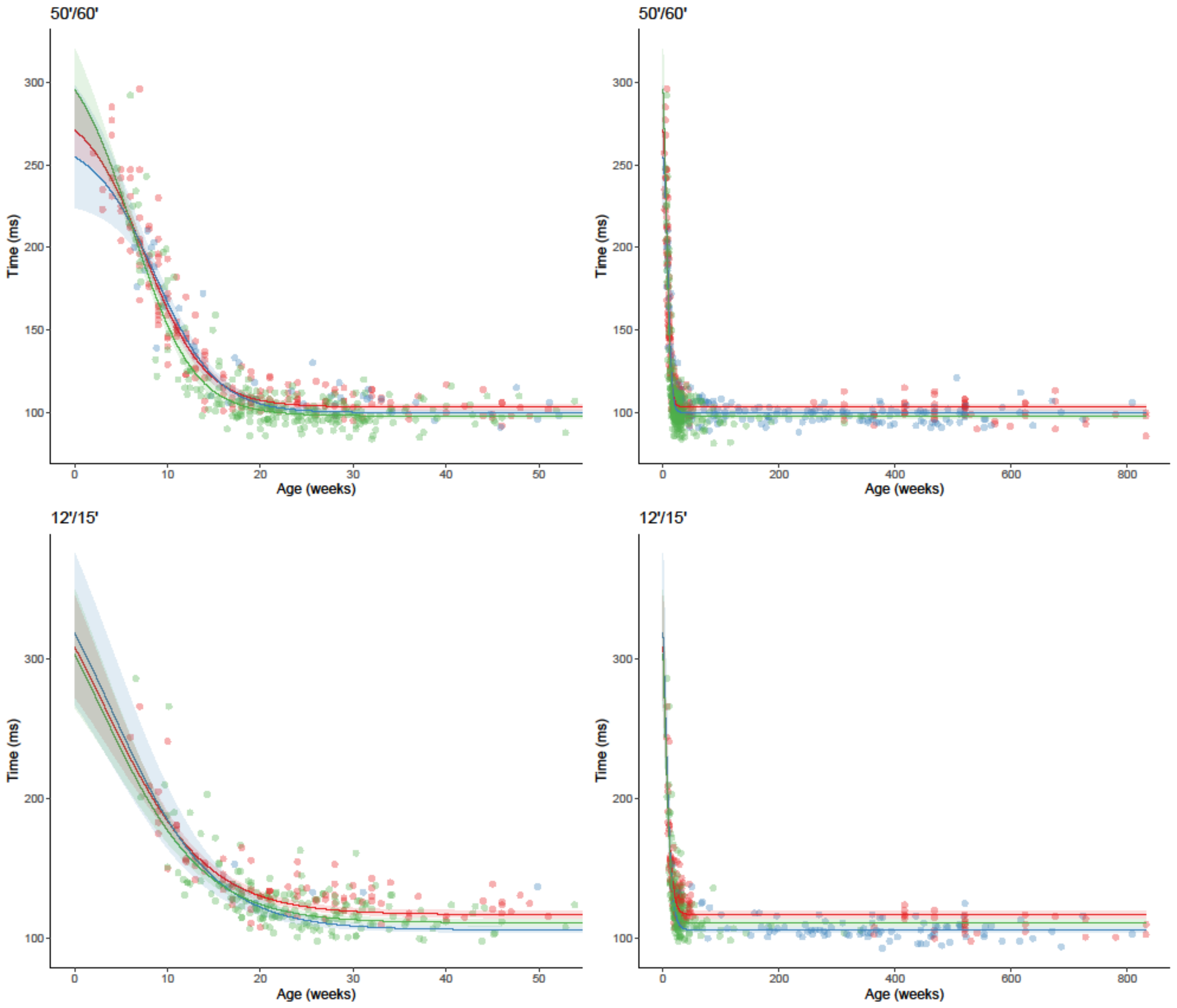

Figure S1. Peak times for large (upper panels) and small (lower panels) by centre. Left hand panels show data up to 52 weeks, right hand panels show all data. Lines regressed medians by centre with coloured shading indicating 90% confidence intervals of the median. Red, GOSH. Blue, RHCG. Green, UPMS.

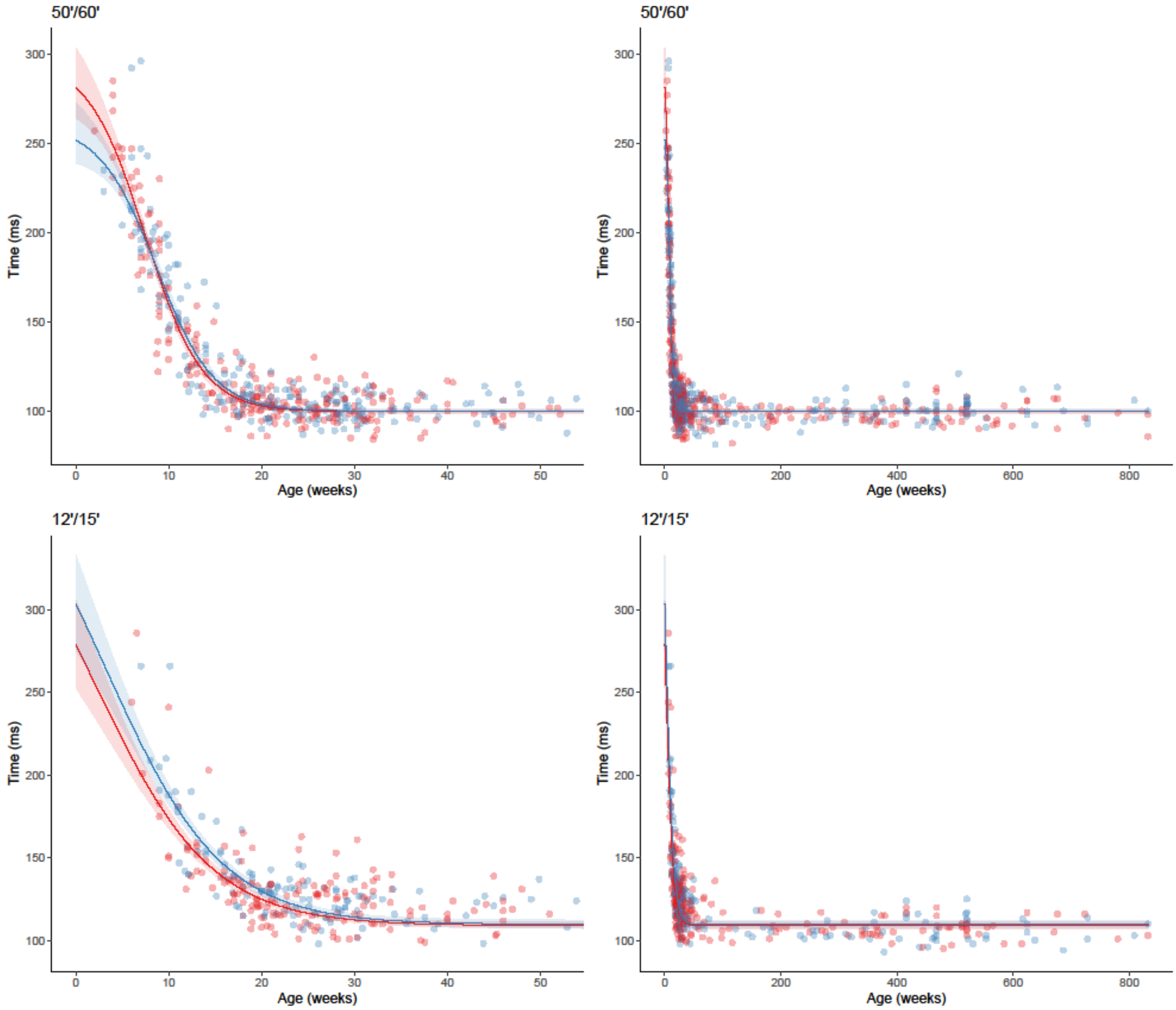

Figure S2. Peak times for large (upper panels) and small (lower panels) by sex for all three centres. Red, female. Blue, male. Left hand panels show data up to 52 weeks, right hand panels show all data. Lines are regressed medians by sex with coloured shading indicating 90% confidence intervals.

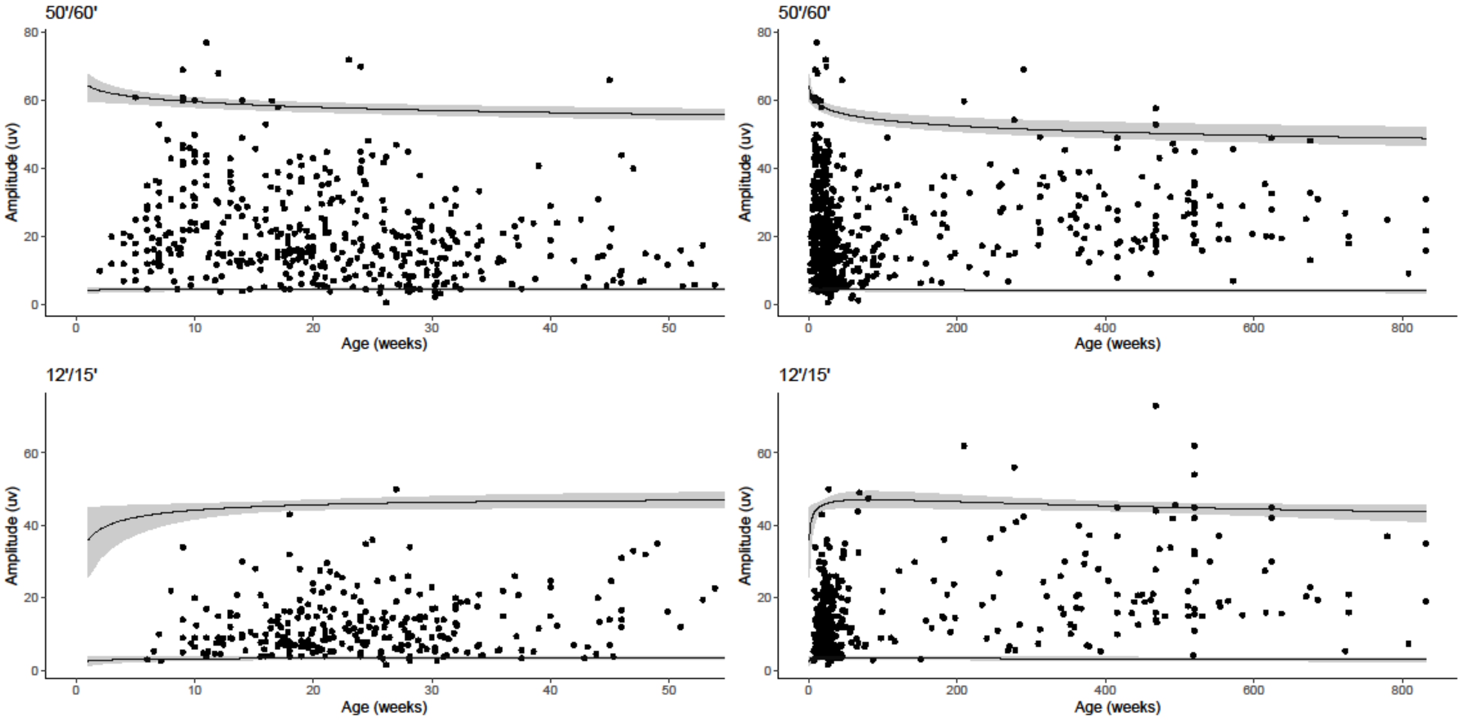

Figure S3. Amplitude data from all three centres and for boys and girls for large checkwidths (upper panels) and small checkwidths (lower panels). Left hand panels show data up to 52 weeks, right hand panels show all data. Lines are regressed upper (97.5) and lower (2.5) percentiles with shading indicating 90% confidence intervals.

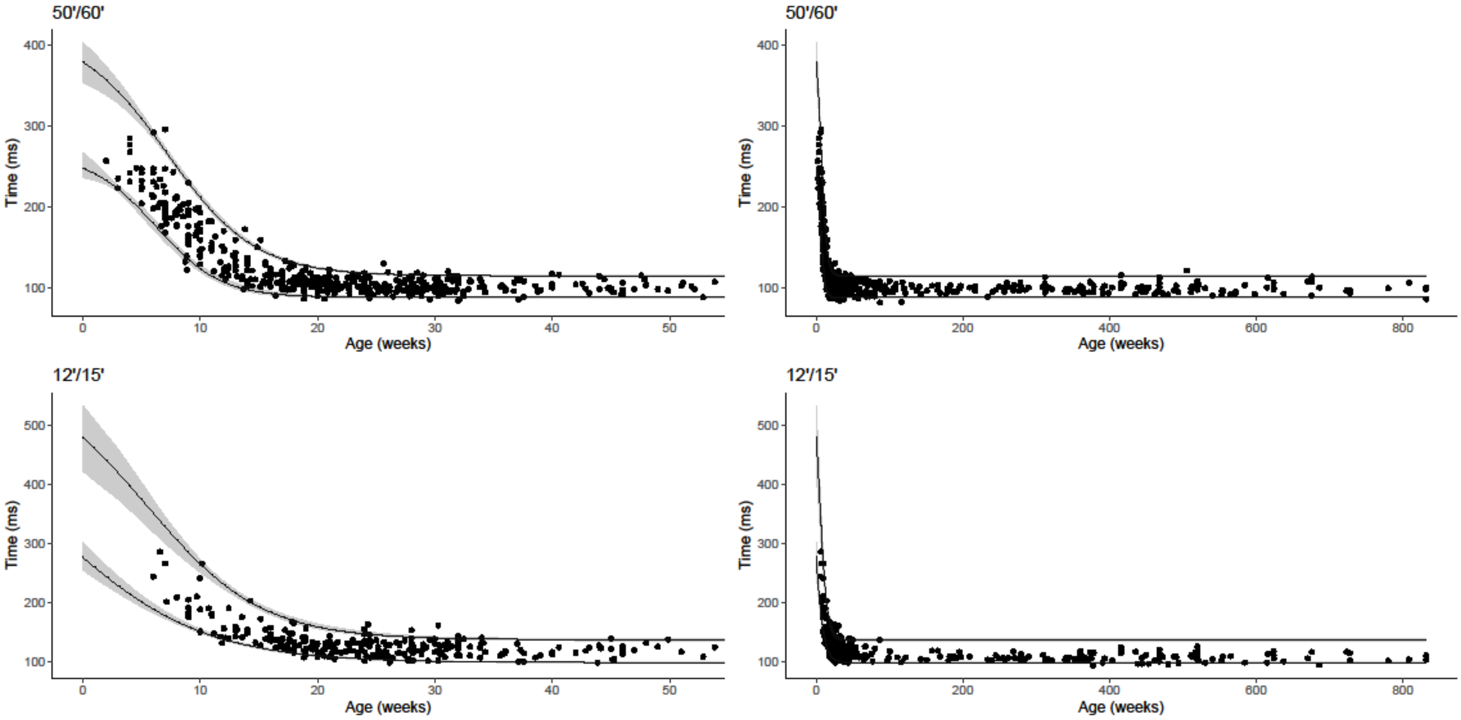

Figure S4. Peak time data from all three centres and for boys and girls for large checkwidths (upper panels) and small checkwidths (lower panels). Left hand panels show data up to 52 weeks, right hand panels show

all data. Lines are regressed upper (97.5) and lower (2.5) percentiles with shading indicating 90% confidence intervals.

Table S1: values for parameters in Equation 2 to calculate lower (2.5th centile) and upper (97.5th centile) amplitude reference limits for large (60’) and small (15’) checkwidths.

		a	b	c	d	e	f	g	h
large checkwidth	lower percentile	-0.02	-0.41	1.49	4.635	3.743	649	1251.8	2215.5
	upper percentile	-0.08	-1.50	4.03					
small checkwidth	lower percentile	-0.49	-0.38	1.20	4.963	3.968	403	723.27	668.2
	upper percentile	-0.37	-0.04	3.82					

Table S2: values for parameters in Equation 3 to calculate lower (2.5th centile) and upper (97.5th centile) peak time reference limits for large (60’) and small (15’) checkwidths.

		a	b	x_0	μ
large checkwidth	lower percentile	87.69	270.55	6.03	-2.80
	upper percentile	114.49	418.53	7.22	-3.74
small checkwidth	lower percentile	96.34	542.72	-1.55	-5.77
	upper percentile	136.83	599.85	5.22	-4.96
